# Imaging evaluation of patellofemoral joint instability: a review

**DOI:** 10.1186/s43019-023-00180-8

**Published:** 2023-03-13

**Authors:** Roberto M. Barbosa, Manuel Vieira da Silva, Carlos Sampaio Macedo, Cristina P. Santos

**Affiliations:** 1 Center of MicroElectroMechanical Systems (CMEMS), University of Minho, Guimarães, Portugal; 2 MIT Portugal Program, School of Engineering, University of Minho, Guimarães, Portugal; 3 LABBELS - Associate Laboratory, Braga/Guimarães, Portugal; 4 Department of Orthopaedics, Trofa Saúde Braga Centro Hospital, Braga, Portugal; 5 Department of Radiology, Trofa Saúde Braga Centro Hospital, Braga, Portugal; 6 Clinical Academic Center (2CA-Braga), Hospital of Braga, Braga, Portugal

**Keywords:** Anterior knee pain, Patellofemoral instability, Trochlear dysplasia, Patellar height, Tibial tubercle lateralization, Diagnostic imaging

## Abstract

The multifactorial origin of anterior knee pain in patellofemoral joint disorders leads to a demanding diagnostic process. Patellofemoral misalignment is pointed out as one of the main causes of anterior knee pain. The main anatomical risk factors of patellofemoral instability addressed in the literature are trochlear dysplasia, abnormal patellar height, and excessive tibial tubercle–trochlear groove distance. Diagnostic imaging of the patellofemoral joint has a fundamental role in assessing these predisposing factors of instability. Extensive work is found in the literature regarding the assessment of patellofemoral instability, encompassing several metrics to quantify its severity. Nevertheless, this process is not well established and standardized, resulting in some variability and inconsistencies. The significant amount of scattered information regarding the patellofemoral indices to assess the instability has led to this issue. This review was conducted to collect all this information and describe the main insights of each patellofemoral index presented in the literature. Five distinct categories were created to organize the patellofemoral instability indices: trochlear dysplasia, patellar height, patellar lateralization, patellar tilt, and tibial tubercle lateralization.

## Introduction

Patellofemoral joint (PFJ) disorders require a demanding diagnosis due to the multifactorial origin of anterior knee pain and the complex interplay of multiple anatomical structures. Besides its multifactorial etiology, anterior knee pain is mainly caused by the abnormal PFJ morphology, resulting in a dysfunction in the extensor mechanism [1, 2]. Other causes include PFJ arthritis, extensor tendinopathy, and ligament and meniscus injuries [3].

The patella has a crucial role in the PFJ biomechanics, which allows centralization of the forces provided by the quadriceps muscle group and increases the moment arm [4, 5]. Patellar tracking comprises the movement of the patella that begins to engage in the trochlear groove at about 20° of flexion [6–8]. During lower degrees of flexion, only soft tissue stabilizers act against lateral forces, and 60% of the restraining forces are provided by the medial patellofemoral ligament (MPFL). For knee flexion higher than 30°, the stability of the patella is ensured mainly by the osseous morphology of the distal femur [4–6, 8]. The pressure in the subchondral bone is increased with an abnormal patellar tracking, leading to knee pain, chondral injuries, and patellofemoral osteoarthritis (PFOA) development, with more expressive complications in patients with recurrent episodes of patellar dislocations [4, 5, 9].

PFJ imaging has a crucial role in revealing the origin of the anterior knee pain and has a significant impact on the individualized pathology management for each patient [10]. Three main anatomical predisposing factors for patellofemoral instability (PFI) are presented in the literature, which are detectable by PFJ imaging: trochlear dysplasia, abnormal patellar height, and an excessive tibial tubercle–trochlear groove (TT–TG) distance [11–19]. There is little consensus regarding the assessment of PFI and, consequently, there is no established protocol for this type of PFJ study. Several approaches have been described in the literature, addressing different PFI indices to quantitatively assess the main risk factors and signs of this pathology, resulting in scattered information and redundant approaches.

This review was conducted with the aim of collating all the PFI indices found in the literature, and describing their methodology and the main insights provided by the authors, including imaging modalities, the image acquisition process, instructions to perform the indices measurements, adequate anatomical landmark positioning and slice image selection to perform the measurements, reference values, and the advantages and limitations, when applicable, contributing to the radiology and orthopedics fields. The purpose of this study was not to identify the most reliable indices, but rather to address all different approaches by the authors for assessing the main risk factors of PFI.

## Patellofemoral instability imaging

PFI assessment goes beyond the PFJ. Some secondary risk factors regarding the overall alignment and torsion of the lower limbs also influence the PFJ stability. Varus and valgus misalignment, genu recurvatum, pathological femoral and tibial torsion angle, patellar dysplasia, and abnormal pronation of the subtalar joint are secondary risk factors that affect the stability of the PFJ [11].

PFJ imaging has undergone considerable evolution over time due to its complexity. Both static and dynamic imaging techniques have been utilized to assess the PFJ. While static imaging allows evaluation of the PFJ morphology, dynamic imaging allows an assessment of its kinematics and the real-time interplay of soft tissues and bony constraints. Dynamic imaging includes the acquisition of images during knee extension and flexion [5]. The studies carried out regarding PFI have demonstrated that the degree of knee flexion and the quadriceps muscles contraction influence the patellar misalignment. Nevertheless, dynamic imaging does not have a well-defined clinical application, and there is no consensus about measurement protocols and reference range values, although it is a promising procedure for better PFJ kinematics assessment [20]. Different approaches are found in the literature aiming to assess PFI in static imaging, including different flexion degrees of the knee and stress-testing of the patella using external forces and active muscle contraction [5, 20, 21].

Standard lateral views are used in plain radiographs to assess the patellar height. The Merchant view is widely used in radiography to assess the morphology of the trochlea and the patellar tilt and lateralization. This is obtained with the patient in the supine position, with the knees at 45° of flexion, and the x-ray beam inclined downward 30° [5, 22].

For a more detailed analysis of the PFJ morphology, cross-sectional studies are more adequate since they provide a more complete assessment. Computed tomography (CT) is the medical imaging technique most used in the literature for PFI diagnostic imaging. However, recent studies are showing great acceptance of using magnetic resonance imaging (MRI) in PFI studies. This enables the analysis of the cartilaginous PFJ surface instead of subchondral bone as in conventional CT or radiography [11, 20]. The trend in this kind of study is to uniformize the PFI diagnosis using MRI. In addition to measurements of PFI indices, it has been clinically demonstrated that MRI is also highly sensitive for detecting capsular, ligamentous, cartilaginous, and bony lesions associated with patellar dislocation events, making it the accepted standard practice [12, 23, 24].

Considering static imaging techniques using radiographs, CT, or MRI, several quantitative indices are measured to assess PFI. They have a crucial role in the detection of the main risk factors of PFI, and some recent studies have addressed the importance of PFI indices to predict PFOA development and progression. Different studies have suggested that almost all PFI factors have a significant contribution to PFOA, including patella alta, trochlear dysplasia, patellar tilt, and tibial tubercle lateralization [25].

The following sections contain all the indices found in the literature for assessing PFI, which can be grouped into five categories: trochlear dysplasia, patellar height, patellar lateralization, patellar tilt, and tibial tubercle lateralization.

## Trochlear dysplasia

**Fig. 1:**
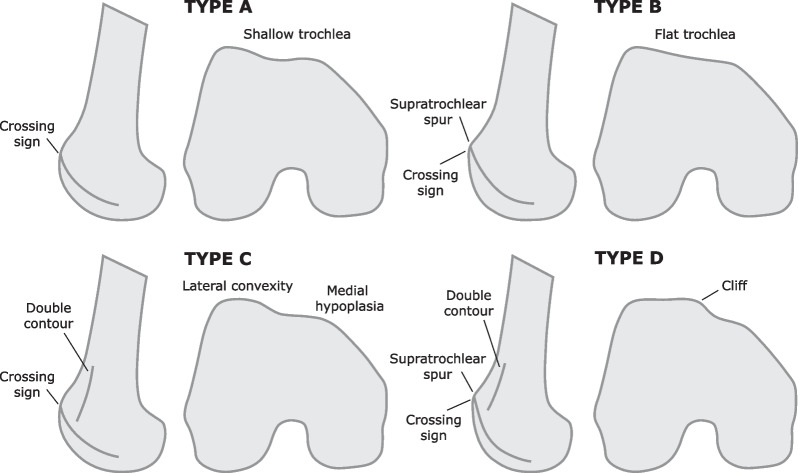
Illustration of the trochlear dysplasia types according to the Dejour classification

Trochlear dysplasia is the main predisposing factor for PFI, with an incidence of 85–96% in patients with recurrent PFI [11, 14–19]. According to the Dejour classification, there are four different types of trochlear dysplasia identified in radiographic images from lateral views with a perfect superimposition of the posterior condyles [15]. This classification system is based on three dysplastic signs that characterize each type of trochlea, as illustrated in Fig. 1. Each type is characterized as follows:

- Type A: the trochlea presents crossing sign in the lateral view. It is concave and symmetrical but is shallower than normal.
- Type B: the presence of crossing sign and supratrochlear spur. The trochlea is prominent and flat in axial images.
- Type C: the presence of crossing sign and double contour. In axial view, the lateral facet is convex and the medial facet is hypoplastic. There is no prominence.
- Type D: the trochlea contains all the previously mentioned signs: the crossing sign, supratrochlear spur, and double contour. There is evidence of a clear asymmetry between the medial and lateral facets.

Regardless of the trochlear type, which influences the approach to correct the dysplasia, a thorough analysis of the trochlear morphology is required. Five PFI indices for assessing the trochlear dysplasia were found in the literature: sulcus angle (SA), trochlear facet asymmetry (TFA), lateral trochlear inclination (LTI), trochlear groove depth (TGD), and ventral trochlear prominence (VTP).

Using cross-sectional studies, SA, TFA, and TGD are measured on the same slice, located 3 cm above the femorotibial joint line, which includes the deepest aspect of the intercondylar groove with an appearance of a Roman arch, where the trochlear surface is completely exposed [20, 26–29].

**Fig. 2:**
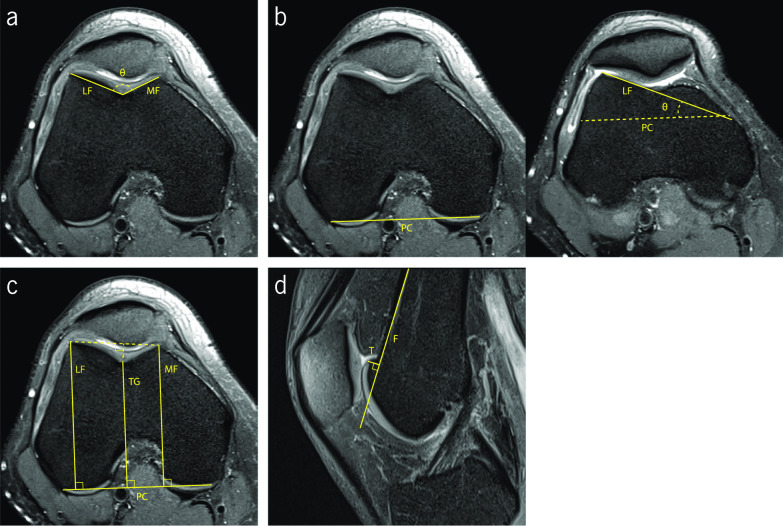
Fat-saturated proton density-weighted magnetic resonance images demonstrating the measurements of the indices to assess trochlear dysplasia. **a** Sulcus angle (∠θ) and trochlear facet asymmetry (MF/LF). **b** Lateral trochlear inclination (∠θ). **c** Trochlear groove depth ((MF + LF)/2–TG) (An alternative method is presented by the dashed line). **d** Ventral trochlear prominence (length T)

SA was first measured using plain radiographs, and then using CT images. Diederichs et al. state that MRI images provide more accuracy and reproducibility for measuring SA. This gives the possibility of using the articular cartilage or subchondral bone for the measurements. Some authors refer to the articular cartilage as the more relevant surface to use in the measurement since it is, in fact, the actual joint surface [28, 29]. However, some studies have been conducted measuring SA in the proximal trochlea, which corresponds to the first craniocaudal image showing the complete trochlear surface [30]. SA corresponds to the angle between the lines that define the lateral and medial trochlear facets (Fig. 2a). The reference values for SA are well-documented in the literature, with a cut-off value of 145°. Higher values are present in patients with pathological flattening [24, 30]. Several studies have been conducted using MR images to measure SA. Osman et al. presented their study with 38 patients with lateral patellar dislocation and 38 control patients. The image acquisition protocol comprised knees in neutral position and the patients in supine position. They obtained a value of 152.7 ± 7.4° for the pathologic group and 134.4 ± 4.1° for the control group [29]. Another study conducted by Charles et al. obtained 137.57 ± 0.93° in the control group with 81 knees and 155.33 ± 1.98° for 40 knees of patients with recurrent patellar dislocations [30]. When using the proximal trochlea to perform the measurements, the values were higher, with a value of 148.48 ± 0.94° in the control group and 165.57 ± 2.65° in the pathological knees [30]. The results show that the measurement of SA performed on the distal trochlea image are closer to the well-documented cut-off value of 145° [24, 30].

TFA is the ratio between the length of medial and lateral trochlear facets (Fig. 2a). TFA values less than 40% are indicative of trochlear dysplasia [26]. Pfirrmann et al. conducted a study using MRI images that demonstrated that the most accurate measurement of TFA was performed in the axial slice located 3 cm above the femorotibial joint space. Using the cut-off value of 40%, their results presented a sensitivity of 100% and a specificity of 96% for detecting the presence of trochlear dysplasia with TFA index [26].

Two different approaches were identified in the literature to measure TGD. One of them resorts to a tangential line connecting the posterior aspect of the femoral condyles as reference. Perpendicular to this line, three lines are drawn: connecting the most anterior aspect of the medial and lateral trochlear facets, and the last one connecting the deepest point of the trochlear sulcus, as shown in Fig. 2c. Sanders et al. refer to a simpler method that uses a line connecting the anterior aspect of both trochlear facets as reference, and another perpendicular line that connects the deepest point of the trochlear groove, as shown in Fig. 2c with a dashed line [31]. A value lower than 3 mm indicates dysplasia, for both methods described [3, 12, 20, 26, 28, 32]. Pfirrmann et al. in their studies obtained a sensitivity of 100% and a specificity of 96% for detecting trochlear dysplasia, resorting to MRI modality and considering the documented cut-off value of 3 mm [26].

LTI was firstly evaluated by resorting to plain radiographs; however, with the evolution of MRI, this has rapidly become the standard method due to its advantage of enabling the identification of the cartilage. The measurement is performed on two axial slices. On the slice 3 cm above the femorotibial joint line, a tangential line that connects the posterior aspect of both femoral condyles is drawn. On the first craniocaudal image showing trochlear cartilage, another line is drawn passing through the subchondral bone of the lateral trochlear facet. LTI is obtained by the angle between both lines (Fig. 2.b). An angle inferior to 11° indicates trochlear dysplasia. Carrilon et al. have conducted a study aiming to establish the correlation between LTI and PFI. LTI was measured in 30 patients with PFI and 30 patients in the control group. The protocol of image acquisition included the full extension of the knee. The results presented a mean value of 6.17 ± 4.97° for patients with PFI and 16.93 ± 4.76° for the control group. Using 11° as the cut-off value, this study showed a sensitivity of 93% and an overall accuracy of 90%. An advantage of LTI is that it considers the proximal portion of the trochlea, a region prone to dysplasia, when the patella is not yet engaged [33].

Finally, VTP is the perpendicular distance between the most ventral cortical point of the trochlear groove and the line parallel to the ventral cortical surface of the distal part of the femur, measured on the mid-sagittal slice (Fig. 2d). There is no consensus regarding the reference value for VTP. Dejour et al. concluded that the most adequate pathologic threshold value is 3 mm, using radiograph studies of true lateral view of the knee. Sixty percent of the knees with objective PFI presented a VTP of 3 mm or more [34]. According to their results, resorting to mid-sagittal plane from MRI studies, Pfirrmann et al. suggested a cut-off value of 8 mm, and more recently, Bollier et al. indicated a value of 4 mm in lateral radiographic view [18, 26].

## Patellar height

Patella alta, also known as high-riding patella, is considered another main factor of PFI, which is present in 25–30% of patients with acute patellar dislocations [12, 35]. Patella alta is characterized by an abnormal position of the patella in relation to the trochlear groove. An excessive length of the patellar tendon can be the origin of this anatomic morphology [36]. Compared with a patient with normal patellar height, in patients with patella alta, a higher degree of knee flexion is necessary for the patella to engage in the trochlear sulcus [12]. In the first degrees of knee flexion, the patellar contact area is reduced, leading to a decrease in stability. MRI is more sensitive to assess the patellar height due to the reliability of the measurement of the patellar tendon [12].

Five PFI indices have been described in the literature to evaluate the patellar height: Insall–Salvati Index (ISI); Modified Insall–Salvati Index (MISI); Caton–Deschamps Index (CDI); Blackburne–Peel Index (BPI); and Patellotrochlear Index (PTI). In cross-sectional studies, all measurements are performed in the sagittal slice showing the longest axis of the patella and the insertion of the patellar tendon in the anterior tibial tuberosity.

**Fig. 3:**
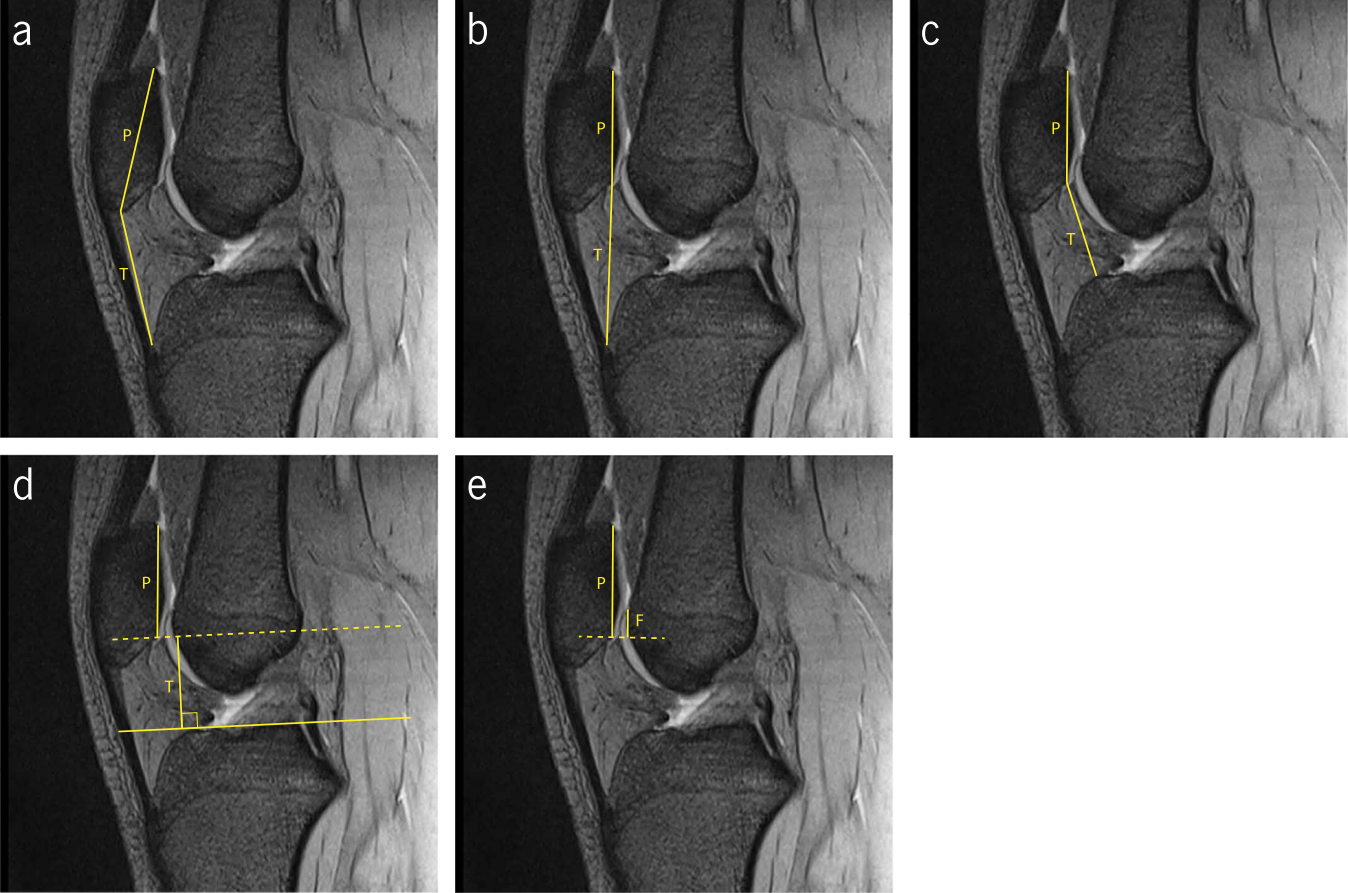
Sagittal gradient echo T2-weighted magnetic resonance images demonstrating the measurements of the indices to assess patellar height. **a** Insall–Salvati Index (T/P). **b** Modified Insall–Salvati Index (T/P). **c** Caton–Deschamps Index (T/P). **d** Blackburne–Peel Index (T/P). **e** Patellotrochlear Index (F/P)

ISI was first described by Insall et al. using the lateral radiographic view with the knee flexed 30° [37]. ISI is obtained by the ratio between the length of the patellar tendon and the length of the patella, from pole to pole (Fig. 3a). Normal patellar height is situated between 0.8 and 1.2 [37]. A value of the ISI smaller than 0.8 is indicative of patella baja and a value greater than 1.2 is indicative of patella alta. Special attention must be considered regarding the morphology of the patella. Abnormal patellar morphologies, such as Grelsamer type II patella, which presents a long distal nonarticulating facet, can lead to abnormal patellar heights being undetectable [7, 19, 24, 37–39]. Lee et al. investigated whether the documented cut-off value for ISI could be applied to both CT and MRI modalities [40]. With the knee in full extension in CT, and the patient in supine position in MRI, they concluded that the cut-off values for these imaging modalities must be slightly adjusted. They propose a cut-off value of 1.33 for MRI and 1.3 for CT [40]. Several studies have corroborated this increment in the MRI modality but with a slightly different amount. A cut-off value of 1.3 for ISI measurements in MRI modality is given by some authors [12, 13, 20, 41]. Miller et al. have highlighted that this index is independent of the degree of knee flexion [41].

MISI was proposed by Grelsamer et al. to improve the sensitivity regarding the patellar morphology when compared with ISI. MISI consists of the ratio between the distance from the patellar tendon insertion on the tibia to the distal end of the patellar cartilage, and the length of the articular surface of the patella (Fig. 3b). The cut-off value that indicates patella alta is 2 [42]. The study was conducted using lateral radiographs. With this modification, Grelsamer et al. showed that 50% of the cases with patella alta would not be detected if only ISI was measured. MISI provides a significant complement for patellar height assessment [42]. Seil et al. state that this method presents a disadvantage compared with the ISI, which is the inherent difficulty in identifying the distal end of the patellar articular surface [39].

CDI is calculated by the ratio between the distance of upper limit of the tibia to the distal end of the patellar cartilage, and the length of the articular surface of the patella (Fig. 3c). CDI values greater than 1.2 indicate patella alta, and values lower than 0.6 indicate patella baja [13]. This index is widely used for planning tibial tubercle osteotomies and to assess the patellar height after high tibial or tibial tubercle osteotomies [11, 13, 20, 32]. Furthermore, it allows assessing the patellar height in different degrees of knee flexion and different sizes, in patellae with pole abnormalities, and in variable skeletal maturation [11, 13, 20, 32]. Concerning the limitations of this index, it is complex to clearly detect the patellar articular surface margins and the upper limit of the tibia, and it gets worse in osteoarthritic knees [13]. Originally measured on plain radiographs, several studies have proved its reliability on MRI, providing better accuracy in the detection of the margin of patellar articular surface [11, 20]. The greatest length of the patellar articular surface must be visible in the slice to perform the measurement [32]. Escala et al. have suggested a cut-off value of 1.1 in a work carried out with MRI for assessing the PFJ morphology [27].

BPI was originally proposed by Blackburne et al. for assessing patellar height in lateral radiographs of the knee flexed 30°. The first step consists in drawing a tangential line to the tibial plateau. BPI is calculated by the ratio between the perpendicular distance from this line to the inferior margin of the patellar cartilage, and the length of the patellar articular surface (Fig. 3.d). A normal value for BPI is 0.8 [43]. Values higher than 1.0 indicate patella alta, and values lower than 0.5 indicate patella baja [43]. Lee et al. propose a cut-off value of 1.09 on MRI [40]. However, it is difficult to measure this PFI index in cross-sectional studies, such as CT or MRI, because a non-flat tibial plateau is often observed in the sagittal section [44].

PTI was proposed by Biedert et al. to assess the patellar height, considering the real articular cartilage relationship in the PFJ on sagittal MRI [45]. The images are acquired with the knee in full extension, the foot in 15° of external rotation, and the quadriceps muscle relaxed. In the literature it is referred to as the more accurate parameter that reveals the functional patella height. To measure this index, the length of the patellar articular surface is firstly identified. A parallel line is traced from the superior margin of the trochlear articular cartilage to the line perpendicular to the first one, started from the inferior margin of the patellar articular surface (Fig. 3e). PTI is obtained by the ratio between the trochlear articular surface and the patellar articular surface. Values higher than 50% indicate patella baja, and values lower than 12.5% indicate patella alta [12, 20, 45].

## Patellar lateralization

Patellar misalignment comprises the translation of the patella laterally. Axial images of the knee are crucial to evaluate the patellar lateralization. There is a significant amount of scattered information regarding this topic. Several PFI indices are listed in the literature for assessing the lateralization of the patella, including: the congruence angle (CA), the patella–lateral condyle (PLC), the lateral shift (LS), the bisect offset ratio (BO), lateral patellar displacement (LPD), patellar displacement (PD), lateral patellofemoral length (LPL), the tangent offset (TO), the lateral patellar edge (LPE), and patellofemoral axial engagement (PAE). All listed PFI indices are measured on two axial slices: the slice 3 cm above the femorotibial joint line, which includes the deepest aspect of the intercondylar groove with an appearance of a Roman arch, and the axial slice with the longest axis of the patella.

**Fig. 4:**
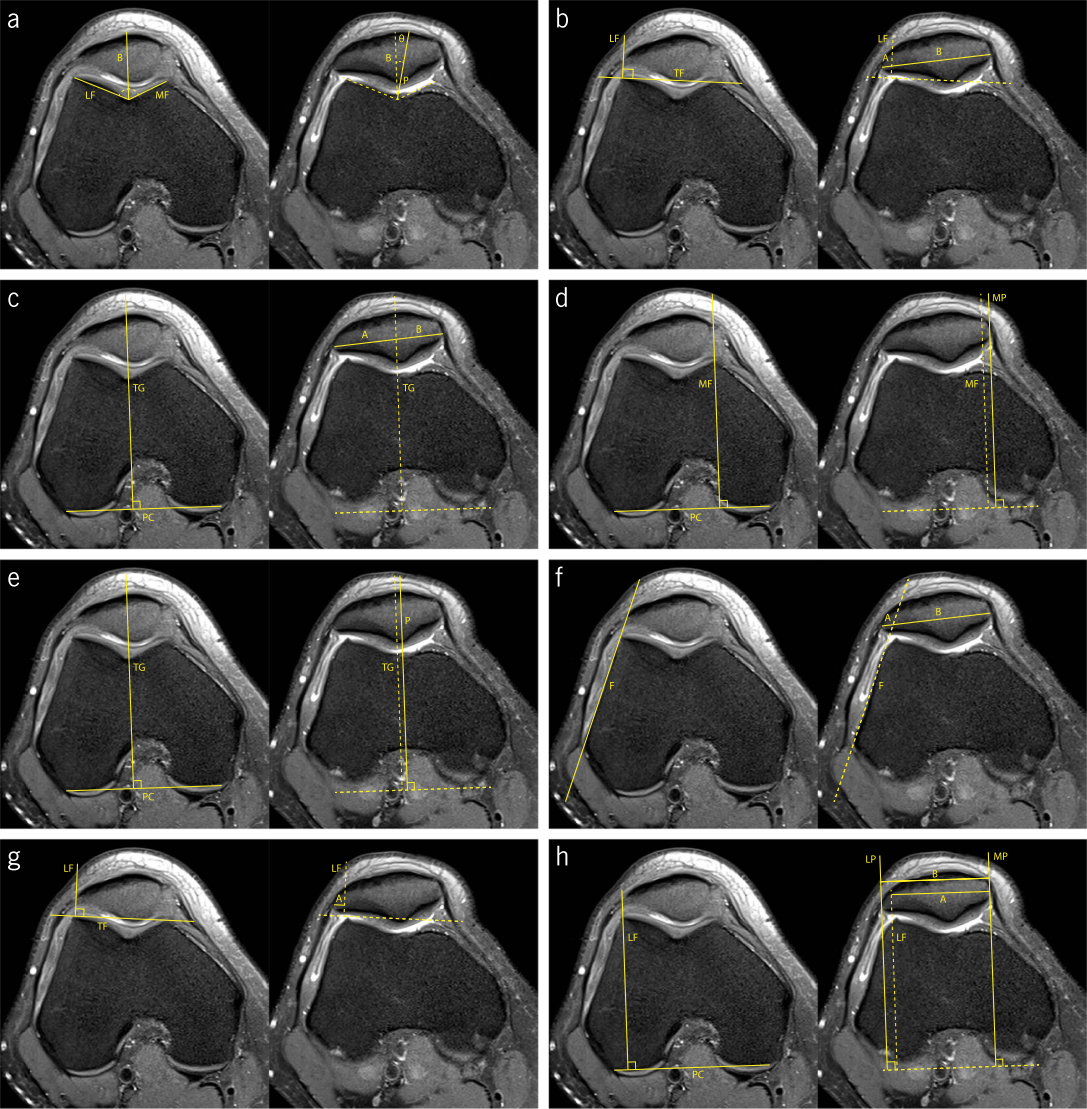
Fat-saturated proton density-weighted magnetic resonance images demonstrating the measurements of the indices to assess patellar lateralization. **a** Congruence angle (∠θ). **b** Patella–lateral condyle [A/(A + B)] and lateral shift (A/B). **c** Bisect offset ratio [A/(A + B)]. **d** Lateral patellar displacement (distance MF–MP). **e** Patellar displacement (distance TG–P). **f** Lateral patellofemoral length (length A) and tangent offset (A/(A + B)). **g** Lateral patellar edge (length A). **h** Patellofemoral axial engagement index (A/B)

CA corresponds to the angle between the bisector of SA, and the line that connects the deepest point of the trochlear groove and the ridge of the patella (Fig. 4a). Using the SA bisector as reference, the CA is negative if it is medial and positive if it is lateral. This was first assessed in plain radiographs employing the Merchant view, where its mean value for normal knees is −6° [46]. Values higher than 16° are associated with lateral patellar subluxation [31, 46, 47]. Aglietti et al. conducted a study that suggests a new cut-off value of 4° and indicates a mean value for normal knees of −8° [48]. Studies addressing the measurement of CA in CT slices are found in the literature, reporting a normal CA value of 0° for this imaging modality. It is suggested that CT images are acquired with the knee at 10° of flexion [31, 49].

PLC and LS are two similar PFI indices to evaluate the patellar lateralization. Both use a line tangential to the anterior aspect of the trochlear facets as reference and another perpendicular line starting from the top of the lateral trochlear facet, intersecting the longest axis of the patella (Fig. 4b). LS is obtained by the ratio between the lateral and the medial patellar portions, and the PLC is obtained by the ratio between the lateral portion and the patellar length [50–52]. Sasaki et al. obtained a mean LS value of 14 ± 5.7% with relaxed quadriceps muscle and a mean value of 28 ± 8.5% with contracted quadriceps, in the control group with normal PFJ and the knees in full extension [50]. In the group with patients presenting subluxation of the patella, the values were 31.4 ± 5.9% with relaxed quadriceps muscle and 59.1 ± 20.4% with contracted quadriceps [50]. Martinez et al. affirm that LS is not accurate for quantifying patellar lateralization [10].

PLC was proposed by Kujala et al., aiming to obtain most reliable quantitative information regarding condylar support for the patella [51]. The study included MRI sequences of the knee with quadriceps muscle relaxed and in different degrees of flexion, from full extension up to 30°. The results demonstrated that the most significant information is obtained with the knee in full extension. The authors affirm that the clinical classification is subjective, but it is indicated that a normal condition presents a value up to 30%, between 30% and 50% indicates mild-to-moderate abnormality, and values greater than 50% indicate abnormal values [51, 52].

To measure BO, a line perpendicular to the tangential line to the posterior aspect of both femoral condyles that passes through the deepest point of the trochlear groove, which divides the patella on the lateral and medial sides. BO is obtained by the percentage of the lateral portion of the patella (Fig. 4c). The reference values for BO are between 44% and 66%. Values above 66% are considered abnormal [20, 53, 54].

LPD consists of the distance between the lines passing through the highest point of the medial trochlear facet and the most medial edge of the patella, using the posterior aspect of the femoral condyles as reference (Fig. 4d). Different authors use the anterior aspect of trochlear facets as reference [55, 56]. Considering the medial trochlear facet line, the LPD value is positive if the medial edge of the patella is laterally positioned. As the medial edge of the patella shows great variation, it can lead to defective measurements and, therefore, it is considered less relevant clinically [57]. Normal values are between −5 and 5 mm [57, 58]. Haj-Mirzaian et al. have suggested a cut-off value of 7 mm in their studies using MRI [20].

PD evaluates the distance between the lines passing through the deepest point of the trochlear groove and the ridge of the patella (Fig. 4e). Some authors use the posterior aspect of the femoral condyles as reference and others have used the anterior aspect of the trochlear facets as reference [55, 57, 59]. Normal values range from −5 to 5 mm [57]. Heesterbeek et al. have affirmed considering clinically relevant PD values higher than 4 mm. They also state that PD is the preferred index of orthopedic surgeons because it expresses the relationship between the deepest point of the patellar ridge and the trochlear groove [57]. Schueda et al. presented a study that included patients divided in four different groups: control group, painful patellar syndrome, potential PFI, and objective PFI. The measurements of the PD index were performed with CT images of the knee at 20° of flexion. Their results concluded that PD is one of the parameters most significant to estimate the risk of PFI. According to their results, the risk of dislocation increases for values of PD over 5 mm, and it is even more significant over 10 mm [59].

LPL and TO use the same reference lines for the measurements. A tangential line to the anterolateral aspect of the femur is drawn, dividing the longest axis of the patella (Fig. 4f). LPL corresponds to the distance of the lateral edge of the patella to the reference line. On MRI, Nicolaas et al. obtained a mean LPL value of 0.8 ± 2.9 mm in healthy knees with 30° of flexion and the quadriceps muscle relaxed [60]. TO is obtained by the ratio between the lateral portion and the patellar width. Stanford et al. obtained values of 22.7 ± 12.9% and 10.8 ± 6.7% for TO in CT images of normal knees, when the knee was in full extension and in 45° of flexion, respectively [54].

Duchman et al. used LPE in their research, using the reference line passing through the anterior aspect of both trochlear facets. The LPE value is obtained by the distance between the lateral edge of the patella and the line passing through the highest point of the lateral trochlear facet (Fig. 4g). They obtained a mean LPE value of 3.9 ± 1.8 mm with the knee in full extension, and 2.5 ± 1.9 mm with the knee at 30°, in MRI images of normal knees [53].

PAE was proposed by Guilbert et al. [61]. Using the posterior aspect of both femoral condyles as reference, three lines are drawn passing through the lateral aspect of the articular trochlear facet, the lateral patellar edge, and the medial patellar edge (Fig. 4h). PAE is obtained by the ratio between the distance from the medial edge of the patella to the lateral trochlear facet and the patellar width. In axial MRI images of the knee nearly extended, values close to 1 are considered normal. The control group obtained a mean PAE value of 0.94 ± 0.09 and the objective PFI group 0.84 ± 0.16 [61].

## Patellar tilt

Different approaches have been addressed in the literature regarding assessment of the patellar tilt. In the first instance, the authors focused on the lateralization until the introduction of the concept patellar tilt by Laurin et al. as a form of misalignment [62]. Six different approaches were found in the literature to assess patellar tilt: patellar tilt angle (PTA), lateral patellofemoral angle (LPA), angle of fulkerson (AF), tilting angle (TA), patellofemoral index (PI), and angle of Grelsamer (AG). Two axial slices are considered to evaluate the patellar tilt in most of the indices: the slice 3 cm above the femorotibial joint line, which includes the deepest aspect of the intercondylar groove with an appearance of a Roman arch, and the axial slice with the longest axis of the patella. PI is measured on the slice presenting the thinner articular space, and AG only uses the longest axis of the patella.

**Fig. 5:**
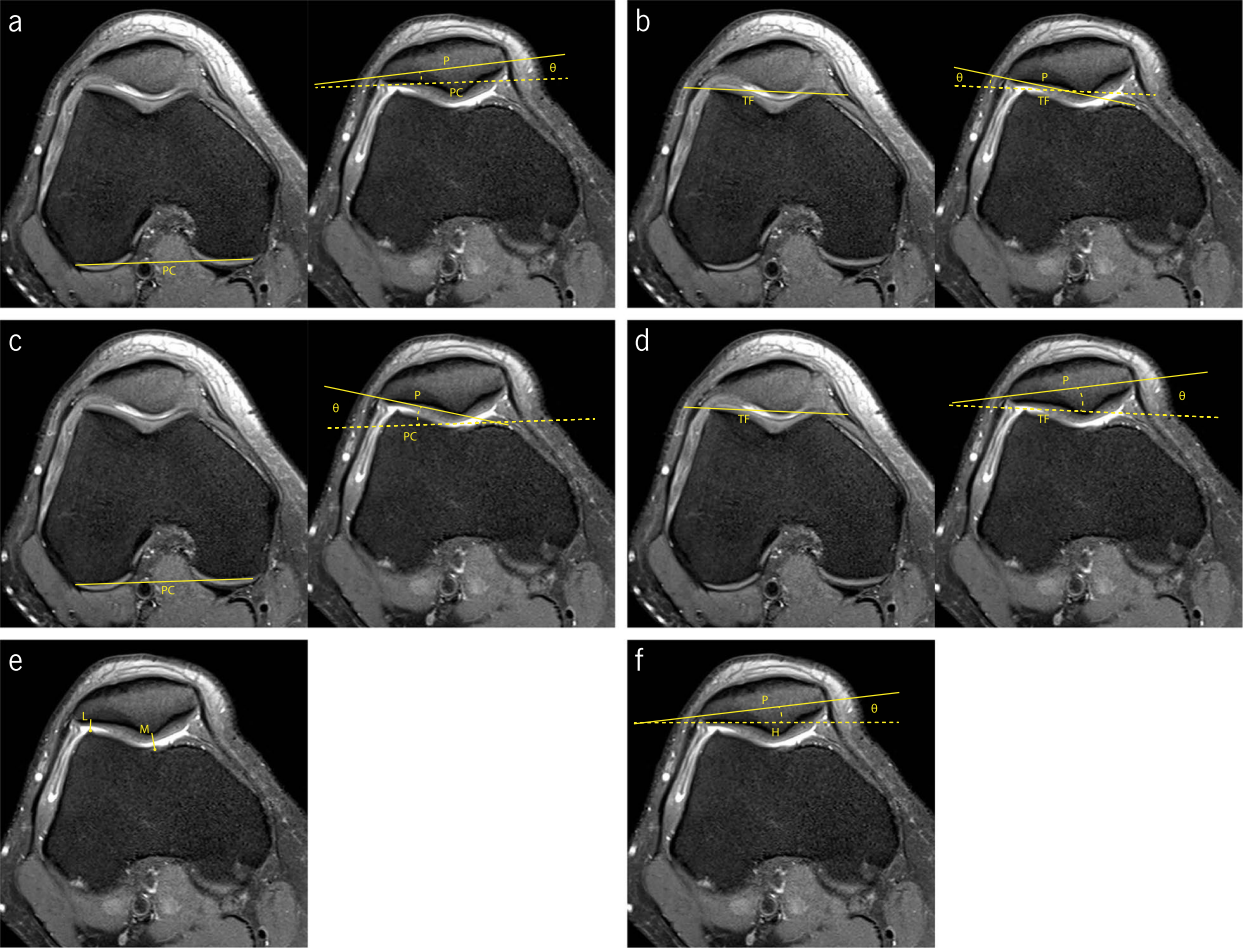
Fat-saturated proton density-weighted magnetic resonance images demonstrating the measurements of the indices to assess patellar tilt. **a** Patellar tilt angle (∠θ). **b** Lateral patellofemoral angle (∠θ). **c** Angle of Fulkerson (∠θ). **d** Tilting angle (∠θ). **e** patellofemoral index (M/L). **f** Angle of Grelsamer (∠θ)

PTA is widely used to assess the patellar tilt. PTA is obtained by the angle between the tangential line to the posterior aspect of both femoral condyles and the line that passes through the longest axis of the patella (Fig. 5a). In the study conducted by Dejour et al. using CT images of the knee in extension, the mean PTA value for the control group was 10 ± 5.8° [34]. The group that included knees from patients with objective PFI presented a value of 28.8 ± 10.5°. This is a simple and reliable measurement and a pathologic threshold for PTA of 20° was suggested, with the quadriceps muscle relaxed. Quadriceps contraction increases the PTA by 1.5° in the control group, and 6° in the objective PFI group, on average [34].

Laurin et al. described the LPA that consists of the angle between a line tangential to the anterior aspect of both lateral and medial trochlear facets and a line tangential to the lateral patellar facet (Fig. 5b). The authors suggest an angle of 20–30° of flexion during image acquisition. The original studies were performed using axial radiographic views. In healthy PFJ, the lines open laterally. If the lines are parallel or they open medially, it is considered an abnormal patellar tilt [62].

AF uses the reference line passing through the posterior aspect of femoral condyles and another line along the lateral facet of the patella (Fig. 5c). It is obtained by the angle formed between both lines [47]. In the studies carried out by Schutzer et al. using CT images of the knee in full extension and with 30° of flexion, AF remained almost constant with the increase of knee flexion in the control group. It had a slight decrease from around 18° to 17° [47]. In the studies conducted by Charles et al. with MRI images of the knee in full extension, the mean values obtained for AF were 18.18 ± 0.56° in the control group and −3.5 ± 2.62° for the pathologic patients, supporting the established cut-off of 8° for pathologic patellar tilt [30, 47].

TA uses the anterior aspect of the trochlear facets as the reference line and the line that passes through the longest axis of the patella (Fig. 5d). TA consists of the angle between both lines. In CT images of the knee in full extension, the control group presented a value of 15.0 ± 4.1° with the quadriceps relaxed and 14.0 ± 1.0° with the quadriceps contracted [50].

PI intends to evaluate the PFJ articular space. Laurin et al. suggested this index, to identify a micro-tilt of the patella, that could be undetectable by LPA [56]. PI is obtained by the ratio between the thickness of the medial and the lateral PFJ interspace. Medial space is defined by the closest distance between the medial trochlear facet and the patellar ridge. Lateral space comprises the closest distance between the lateral aspect of the lateral trochlear facet and lateral patellar facet (Fig. 5e). With the knee flexed at 20° and the quadriceps relaxed, values up to 1.6 are classified normal. A micro-tilt is detected when the values are higher than 1.6 [7, 22, 56].

AG is the simplest method to assess the patellar tilt. Grelsamer et al. considered the angle formed by a horizontal line and the line through the longest axis of the patella (Fig. 5f). The mean value of AG for the control group was 2 ± 2° and for the group containing the pathologic knees was 12 ± 6°. According to the results, the authors suggest a cut-off value of 5°. Values higher than 5° suggest an excessive patellar tilt. Leg rotation during the image acquisition influences the outcome of the measurements. Since it does not use an anatomic reference for the reference line, the measurement results are intrinsically dependent on the leg rotation [63].

## Tibial tubercle lateralization

Bony misalignment of lower limbs has significant relevance in the setting of PFI. Coronal alignment of the lower limbs has begun to be assessed by measuring the Q angle, aiming to evaluate the operating angle of the extensor mechanism. Valgus alignment is often associated with PFI-related problems. Due to the difficulty of quantifying the Q angle, cross-sectional imaging techniques have rapidly become the gold standard method for assessing the behavior of the extensor mechanism. Quantification of the anterior tibial tuberosity lateralization contributes to useful indicators of misalignment. This quantification is relevant to plan surgical distal realignment procedures [64, 65]. Two PFI indices were found for this purpose: tibial tubercle–trochlear groove (TT-TG), and tibial tubercle–posterior cruciate ligament (TT-PCL) distances.

**Fig. 6:**
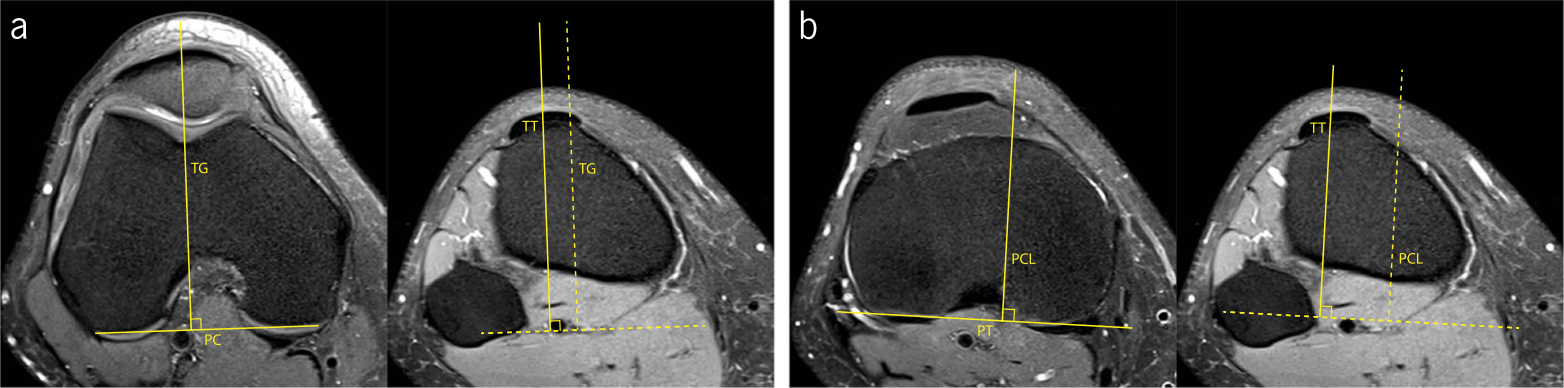
Fat-saturated proton density-weighted magnetic resonance images demonstrating the measurements of the indices to assess tibial tubercle lateralization. **a** Tibial tubercle–trochlear groove distance (distance TT–TG). **b** Tibial tubercle–posterior cruciate ligament distance (distance TT–PCL)

Two axial images are necessary to measure the TT–TG distance: the axial slice 3 cm above the femorotibial joint line that includes the intercondylar groove with an appearance of a Roman arch, and the axial slice with the insertion of the patellar tendon in the anterior tibial tuberosity. A tangential line to posterior femoral condyles is used as reference. TT–TG distance is obtained by the distance between the perpendicular lines that pass through the deepest point of the trochlear groove and the midpoint of the insertion of the patellar tendon in the anterior tibial tuberosity (Fig. 6a). It was originally described for CT images, but recent studies have supported the use of MRI for assessing this parameter [66, 67]. Knee in full extension is suggested for more reliable measurements [64, 68–70]. Some discussion is raised regarding the factors that influence the outcome of the TT–TG distance measurements. Besides the degree of knee flexion, Pennock et al. showed the influence of age, gender, and size of the patient in the TT–TG distance outcomes [68]. Their research has shown an increase of 0.12 in TT–TG distance for each centimeter in patient height [68]. These factors have led to some inconsistencies in the literature regarding the reference values of this PFI index. A cut-off of 15 mm is used by Thakkar et al. [71]. A systematic review conducted by Tan et al. compares the TT–TG distance performed in CT and MRI. The outcome of this study indicates that both medical imaging modalities are reliable for assessing the TT–TG distance, although the cut-off values are different, once the TT–TG distance on CT was significantly greater. With all data collected, they suggest a cut-off of 15.5 ± 1.5 mm on CT and 12.5 ± 2 mm when using MRI to measure the TT–TG distance [67]. Nevertheless, several authors indicate 20 mm as the cut-off value for TT–TG distance [7, 20, 29, 46].

Seitlinger et al. have proposed the TT–PCL distance to evaluate the tibial tubercle lateralization [64]. On the axial slice below the articular surface of the tibia plateau and above the fibular head, a line is drawn tangential to the posterior aspect of the condylar line of proximal tibia, as reference. A perpendicular line is drawn in the medial border of PCL in the most distal axial slice where the ligament could be clearly identified, corresponding to the insertion of the ligament on the tibia. TT–PCL distance is given by the distance between the line passing through PCL and the line passing through the midpoint of the insertion of the patellar tendon in the anterior tibial tuberosity (Fig. 6.b) [64]. A cut-off value of 24 mm was established. Values higher than 24 mm indicate excessive tibial tubercle lateralization [64]. Recent studies have suggested adjustments in the cut-off value for this PFI index, suggesting a value between 20 and 21 mm [65]. Some authors have emphasized that the TT–PCL distance describes the pure lateralization of the tibial tubercle once it is measured, resorting only to anatomical landmarks of the tibia, excluding the influence of the extensor mechanism of the knee joint in the measurements [64, 65].

## Summary

Due to its multifactorial origin, PFI is a complex pathology to diagnose. Herein, the role of imaging is highlighted in the search for the cause of the instability. This review classifies the predisposing risk factors of PFI into five groups: trochlear dysplasia, patellar height, patellar lateralization, patellar tilt, and tibial tubercle lateralization. Trochlear dysplasia has an incidence of up to 96% in patients with recurrent PFI, and it was found significant coherency and acceptance by the medical community regarding its assessment, addressing LTI, TGD, TFA, and SA. VTP was also found to assess the severity of the dysplasia; however, there was no consistency regarding its reference values. Patellar height is also presented as a main factor of instability, being present in 25–30% of the patients with this condition. Several indices are suggested for this assessment. PTI is identified as one of the most reliable indices to measure the patellar height. CDI is widely used for planning and assessing tibial tubercle osteotomies. Given its clinical acceptability and popularity for assessing the patellar height, ISI must be included in the diagnostic process. To assess patellar lateralization and tilt, several approaches are described in this review. In relation to patellar lateralization, PD is one of the preferred methods since it has been presented in the literature as one of the most significant indices to estimate the risk of PFI and as one of the preferred methods by orthopedic surgeons. PTA deserves equal emphasis for patellar tilt assessment since it is widely used in the setting of PFI and due to its reliability and simplicity. TT–TG is widely used for assessing the tibial tubercle lateralization, and it is presented as a reliable method. Therefore, it should be included in knee examinations. As TT–TG distance is directly influenced by the extensor mechanism of the knee, some concerns must be considered regarding the degree of knee flexion and the size of the patient. On the other hand, TT–PCL uses anatomical landmarks of the tibia, and the literature states that it is able to express the true lateralization of the tibial tubercle.

## Conclusions

Diagnostic imaging plays a crucial role in revealing the origin of PFI and, consequently, in delineating the patient-specific treatment. Despite the extensive work that has been presented in the literature with respect to the quantitative assessment of PFI, this process lacks robustness and standardization. The quantitative indices found in the literature to assess the main predisposing factors of PFI have been described along with their appropriate methodologies. This review provides a comprehensive guide for the correct use of PFI indices, which is the initial step towards a proper assessment of PFI. Future work should address the reliability of all PFI indices, helping to achieve a well-established and uniform protocol assessment.

## Data Availability

Not applicable.

## Abbreviations

AF: Angle of Fulkerson
AG: Angle of Grelsamer
BO: Bisect offset
BPI: Blackburne–Peel index
CA: Congruence angle
CDI: Caton–Deschamps index
CT: Computed tomography
ISI: Insall–Salvati index
LPA: Lateral patellofemoral angle
LPD: Lateral patellar displacement
LPE: Lateral patellar edge
LPL: Lateral patellofemoral length
LS: Lateral shift
LTI: Lateral trochlear inclination
MISI: Modified Insall–Salvati index
MPFL: Medial patellofemoral ligament
MRI: Magnetic resonance imaging
PAE: Patellofemoral axial engagement
PD: Patellar displacement
PFI: Patellofemoral instability
PFJ: Patellofemoral joint
PFOA: Patellofemoral osteoarthritis
PI: Patellofemoral index
PLC: Patella-lateral condyle
PTA: Patellar tilt angle
PTI: Patellotrochlear index
SA: Sulcus angle
TA: Tilting angle
TFA: Trochlear facet asymmetry
TGD: Trochlear groove depth
TO: Tangent offset
TT-PCL: Tibial tubercle–posterior cruciate ligament
TT-TG: Tibial tubercle–trochlear groove
VTP: Ventral trochlear prominence
